# Comparison of different green extraction methods used for the extraction of anthocyanin from red onion skin

**DOI:** 10.1002/fsn3.4354

**Published:** 2024-07-21

**Authors:** Nasim Mirzazadeh, Hadiseh Bagheri, Mehdi Mirzazadeh, Somaye Soleimanimehr, Fatemeh Rasi, Sahar Akhavan‐Mahdavi

**Affiliations:** ^1^ Islamic Azad University Pharmaceutical Sciences Branch Tehran Iran; ^2^ Department of Food Science and Technology, Sari Branch Islamic Azad University Sari Iran; ^3^ Department of Food Science and Technology, Faculty of Agriculture, Kermanshah Branch Islamic Azad University Kermanshah Iran; ^4^ Food and Drug Administration (FDA) Kermanshah University of Medical Sciences Kermanshah Iran; ^5^ Department of Food Science and Technology Gorgan University of Agricultural Sciences and Natural Resources Gorgan Iran

**Keywords:** anthocyanidins, anthocyanin, extraction, extraction efficiency, high pressure‐assisted extraction

## Abstract

Green extraction primarily emphasizes developing new extraction techniques that consume less energy. It involves using safe, non‐toxic alternative solvents and sustainable natural resources to ensure the production of safe and high‐quality extracts. Red onion skin is an important source of anthocyanins, a subgroup of phenolic compounds. Anthocyanins are an important group of natural pigments that have attracted a lot of attention due to their health benefits. However, the instability and high sensitivity of these pigments have limited their use in food and cosmetics. Therefore, in this study, various modern green extraction methods were used, including solvent extraction, ultrasound‐assisted extraction, subcritical water extraction, microwave‐assisted extraction (MAE), pulsed electric field extraction, supercritical fluid extraction (SFE), and high hydrostatic pressure‐assisted (HHPAE) extraction, to specifically extract and purify anthocyanins. The extraction efficiency, specifically targeting anthocyanins, showed the highest efficiency with HHPAE (81.84%) and the lowest with MAE (40.01%). Measurement of total anthocyanin content revealed that HHPAE and SFE methods yielded the highest anthocyanin concentrations, with 248.49 and 244.98 mg/L, respectively. Identification of anthocyanin by LC–MS revealed that the main anthocyanidins in red onion peel are pelargonidin, cyanidin, delphinidin, and petunidin. These results indicate that innovative green extraction methods, particularly HHPAE and SFE, can effectively replace conventional techniques due to their superior efficiency and enhanced preservation of anthocyanin compounds.

## Graphical abstract

In this study, novel and green methods were used to extract anthocyanin from red onion skin.

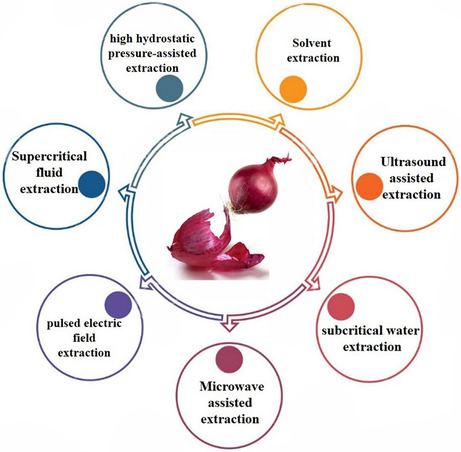

## 1 INTRODUCTION

Color plays a crucial role among the key characteristics influencing a consumer's perception of food quality, as it shapes the initial impression of its edibility. A global shift is observed in favor of employing natural additives overall, with a specific emphasis on food coloring agents. The food industry is increasingly inclined towards adopting natural colorants instead of artificial dyes, driven predominantly by safety concerns. This growing interest necessitates a deeper understanding of the chemical composition and stability of natural pigments, enabling their effective adaptation to the demands of processing, packaging, and distribution (Mahdavi et al., 2014).

Food waste recovery in the food industry is essential for promoting sustainability, resource efficiency, economic viability, and social responsibility. It aligns with broader global efforts to reduce waste and create more sustainable practices within the food supply chain (Yadav et al., 2023). The extraction of natural colorants from food waste aligns with sustainable practices, as it reduces the reliance on synthetic colorants and supports the use of environmentally friendly alternatives. Sustainable practices enhance the overall image and reputation of food companies among environmentally conscious consumers. Moreover, consumers increasingly prefer products that are environmentally friendly and sustainable. Using natural colorants extracted from food waste can enhance the appeal of products to eco‐conscious consumers (Affat, 2021; Bechtold & Mussak, 2009). The issue of food waste poses a significant challenge for the food processing industry, particularly when it entails the loss of valuable nutrients and phytochemicals. The growing consumer demand for processed food emphasizes the need to address this problem by transforming waste into valuable and useful products.

Red onions, varieties of *Allium cepa*, feature a purplish‐red exterior and white interior with red streaks. Primarily utilized in culinary practices, their skins are also employed as natural colorants. Over the last two decades, global onion production has experienced a minimum 25% increase, reaching an annual output of approximately 47 million tons, solidifying its position as the second most significant horticultural crop (Chadorshabi et al., 2022). Primarily utilized for their distinct flavor and potential health benefits, they also serve as a rich source of natural pigments, including anthocyanins. These pigments are predominantly located in the skin of the red onion, making the outer layers a valuable, yet often discarded, resource (Celano et al., 2021).

Anthocyanins, distinguished by their vibrant red, purple, and blue hues in fruits and flowers, are a type of flavonoid with potent antioxidant properties, playing a crucial role in protecting cells from oxidative stress. Their health benefits extend to anti‐inflammatory, anti‐cancer, and anti‐diabetic effects, which have garnered significant interest in the field of nutraceuticals and functional foods. However, their application is limited by their instability, which is influenced by factors such as pH, temperature, and light exposure (Mahdavi, Jafari, Assadpoor, & Dehnad, 2016; Mahdavi, Jafari, Assadpour, & Ghorbani, 2016).

Anthocyanins are typically extracted using polar organic solvents like methanol or ethanol in acidified water to ensure stability. This method effectively breaks down cell structures and releases anthocyanins, which are water‐soluble but require acidic conditions for optimal extraction (Liu et al., 2020; Pereira da Silva et al., 2018). Other specialized techniques, such as ultrasound‐assisted extraction and enzyme‐assisted extraction, further enhance efficiency and preserve anthocyanin integrity by minimizing degradation and reducing solvent usage. In contrast to general phenolic compound extraction, anthocyanin extraction necessitates specific solvent choices, acidic conditions for stability, and careful handling to maintain color intensity and bioactivity, which are crucial for applications in food, pharmaceuticals, and cosmetics (Mahdavi, Jafari, Assadpoor, & Dehnad, 2016; Mahdavi, Jafari, Assadpour, & Ghorbani, 2016).

Developing efficient extraction methods is crucial for the cost‐effective and eco‐friendly extraction of valuable compounds. Green extraction refers to the use of environmentally friendly and sustainable methods for the extraction of bioactive compounds. The concept of green extraction aims to minimize the environmental impact and reduce the use of hazardous chemicals in the extraction process (Zahed et al., 2023).

Modern green extraction methods have been developed to improve efficiency, reduce environmental impact, and maintain the quality of extracted compounds. Table 1 presents the comparison of several such methods. The best method for anthocyanin extraction often depends on the specific source material (such as fruits, vegetables, or plant tissues), the desired purity and concentration of the anthocyanins, and considerations of efficiency, cost, and environmental impact. However, several methods are commonly used and recognized for their effectiveness (Akhavan‐Mahdavi & Mahdi Jafari, 2024; Wang et al., 2020). Each method has its advantages and limitations. The choice of extraction method can also depend on the scale of extraction (laboratory, pilot, or industrial scale), the stability of anthocyanins in different conditions, and the desired application of the extract. Often, a combination of methods is used to optimize yield, purity, and cost‐effectiveness (Pereira da Silva et al., 2018; Silva et al., 2017).

**TABLE 1:** An overview of the features of the extraction methods used in this study.

Methods	Description
Solvent Extraction (SE)	Traditional method involving the use of organic solvents Typically requires a longer extraction time and higher temperatures Potential for solvent residues in the final product
Ultrasound‐Assisted Extraction (UAE)	Uses ultrasonic waves to create cavitation in the solvent, enhancing the penetration and extraction of compounds Faster than SE, with lower temperatures and reduced solvent usage Effective for extracting a wide range of compounds
Subcritical Water Extraction (SWE)	Utilizes water at high temperatures and pressures below the critical point Avoids the use of harmful solvents and is considered environmentally friendly Efficient for extracting heat‐stable and polar compounds
Microwave‐Assisted Extraction (MAE)	Employs microwave energy to heat the solvent and matrix, accelerating the extraction process Quick and efficient, with reduced solvent consumption Suitable for extracting a variety of bioactive compounds
Pulsed Electric Field (PEF) Extraction	Involves applying short bursts of high voltage to disrupt cell walls, enhancing the release of compounds Low thermal impact, preserving heat‐sensitive compounds

So far, various conventional and novel extraction methods have been applied for bioactive compound extraction, among which the most important ones include ultrasound‐assisted extraction (UAE) (Wen et al., 2018), supercritical fluid extraction (SFE) (Uwineza & Waśkiewicz, 2020), subcritical water extraction (SWE) (Zhang et al., 2020), pulse electric field‐assisted extraction (PEFE) (Carpentieri et al., 2023), high hydrostatic pressure‐assisted (HHPAE) extraction (Šeremet et al., 2021), microwave‐assisted extraction (MAE) (Alvi et al., 2022), and pressurized liquid extraction (PLE) (García et al., 2021) and comparison of various extraction methods such as maceration, ultrasonic assisted and supercritical extraction techniques of red onion peel extract (Razavi & Kenari, 2016). Although various studies have been conducted in the field of different methods of extracting bioactive compounds, so far no study has been conducted regarding the extraction of anthocyanin from red onion skin. Therefore, the aim of this study is to compare several new methods of anthocyanin extraction from red onion skin.

## 2 MATERIALS AND METHODS

### 2.1 Materials

Outer dry protective layers of red‐skin onion bulbs (*Allium cepa L*.) were supplied from Kermanshah, Iran. Samples were collected after the harvest and naturally dried, then ground and sieved. The powders were used for extraction. Every other chemical employed in this research was analytical grade and obtained from suppliers of chemicals.

### 2.2 Anthocyanin extraction and purification

To specifically target anthocyanin compounds, we employed various green extraction methods. The extraction procedures were optimized to maximize the yield and purity of anthocyanins, including solvent extraction (SE), ultrasound‐assisted extraction (UAE), subcritical water extraction (SWE), microwave‐assisted extraction (MAE), pulsed electric field (PEF) extraction, supercritical fluid extraction (SFE), and high hydrostatic pressure‐assisted (HHPAE) extraction.

### 2.3 Solvent extraction

The extraction method was modified from Mahdavi, Jafari, Assadpoor, and Dehnad (2016) and Mahdavi, Jafari, Assadpour, and Ghorbani (2016) using a reflux system at the Drugs Research Institute, Shahid Beheshti University, Tehran (Iran). Onion skin was placed into a flask containing a mixture of acidified ethanol and distilled water in a 1:3 ratio. This flask, along with the condenser, was placed in a water bath and heated to 50°C for 2 h. After this period, the flask was removed from the setup and left in a dark place for an additional 2 h. The mixture was then vacuum‐filtered. The resulting extract was concentrated to a density of 15° brix at 40°C using a rotary evaporator (IKA, Germany). The concentrated samples were subsequently stored in brown bottles (Mahdavi, Jafari, Assadpoor, & Dehnad, 2016; Mahdavi, Jafari, Assadpour, & Ghorbani, 2016).

### 2.4 Ultrasound‐assisted extraction

Ten grams of the sample in dried powder form were combined with 90% ethanol in a ratio of 1:10. The mixture was then subjected to ultrasonic treatment using an Elma Transsonic ultrasonic bath model 690/H (Cottbus Germany) at a frequency of 37 kHz for 20 min at 40°C. After sonication, the extract was filtered and later concentrated through the use of a rotary evaporator. The resulting concentrated extracts were preserved at −18°C until further analyses (Egüés et al., 2021; Liu et al., 2020).

### 2.5 Subcritical water extraction

Subcritical water extraction was conducted utilizing a specialized SWE device at the Drugs Research Institute, Shahid Beheshti University, Tehran (Iran). This apparatus comprises a distilled water reservoir, a pump (Comet type: MTP AX 2/70 m) capable of generating pressures up to 170 ± 5 bar, a 140 mL extraction cell with a robust wall designed to withstand high pressure, a heating coil, a pressure gauge, and a temperature control system. Figs were initially ground using a Black & Decker grinder (Model no. JBG60, USA), and the resulting powdered sample was loaded into the extraction cell. The extraction process took place within a temperature range of 110–170°C for durations spanning 10–50 min. To separate impurities from the liquid extract, a vacuum condition and filter paper (Whatman paper no. 4) were employed. The filtered extracts were then cooled and stored in dark polypropylene bags at 4 ± 1°C until utilized in subsequent analyses (Munir et al., 2018).

### 2.6 Microwave‐assisted extraction

Microwave‐assisted extraction was conducted utilizing a microwave cooperative extractor (CW‐2000, Xintuo, Shanghai, China). In this process, a 10.0 g powdered sample was placed into the extractor along with 100 mL of ethanol. The extraction was carried out at 60°C for 30 min, maintaining a constant microwave power of 200 W. The selection of experimental parameters was based on the study by Dong et al. (2021). Following the microwave‐assisted extraction, the solvent was eliminated through vacuum evaporation at 40°C (Dong et al., 2021).

### 2.7 Pulsed electric field extraction

A PEF (Pulsed Electric Field) generator provided by Faraazmoon, Iran, with the capability to generate high‐voltage pulses reaching up to 30 kV and equipped with a 0.25 μF reactor, was employed. The device's treatment chamber was made from Corian, a dielectric material measuring 40 mm in diameter and 16 mm in height, and was fitted with two stainless‐steel caps, a spark gap for managing electrical pulses, along with a pair of electrodes (one stationary and one mobile). The generator was hooked up to both electrodes. Powdered samples were deposited in the chamber. To ensure effective contact between the electrodes and the test material, the chamber was filled with a phosphate buffer solution at a pH of 6.5 before being sealed with the mobile electrode. In the case of pulp samples, the entire 20 cm^3^ of the electrical treatment chamber was meticulously filled with the sample without incorporating any additional solvents. The PEF application was defined by several critical parameters: an electric field strength set at 4.38 kV/cm, a series of 20 pulses, and an energy input quantified at 4.86 kJ/kg (Nowacka et al., 2019; Redondo et al., 2018).

### 2.8 Supercritical fluid extraction

Five grams of samples were introduced into a supercritical fluid extractor (Zhejiang Kingstone, KST‐C002, China). To prevent dispersion effects, 95.0 g of inert glass beads were incorporated to fill the vessel volume. The samples underwent a CO_2_ flow rate of 15 g per minute, and the extraction process was set at a dynamic time of 30 min. These specific operational parameters were optimized for carrot peels using 59.0°C, a pressure of 350 bar, and 15.5% (v/v) ethanol as a co‐solvent (de Andrade Lima et al., 2018).

### 2.9 High hydrostatic pressure‐assisted extraction

Prior to the extraction, assisted by high hydrostatic pressure (HHP), the dried powder was measured and dispersed in 50 mL of deionized water. The mixture was then placed in a stirring water bath at room temperature for 24 h. Following this, the samples were transferred to polyethylene bags, which were vacuum‐sealed after heat‐sealing. The bags containing the water extracts underwent HHP for 17.5 min at 300 MPa. The HHP‐assisted extraction was carried out using a hydrostatic press (Shenzhen Kuanbao, China) with a pressure vessel measuring 200 mm in inner diameter and 2000 mm in length, capable of operating at a maximum pressure of 600 MPa (Rodrigues et al., 2017). Following high hydrostatic pressure (HHP) treatment, the water extracts were removed from the bags and then centrifuged at 5000 ***g*** for 10 min at a temperature of 4°C using a Medifriger BL‐S centrifuge from Selectlab, China at Faculty of Agriculture, Ferdowsi University of Mashhad. The supernatants obtained were then passed through a glass filter funnel, and the filtered extracts were subsequently frozen at −80°C, awaiting the freeze‐drying procedure (Uwineza & Waśkiewicz, 2020).

### 2.10 Extraction efficiency

After every unique extraction process, the solution was subjected to centrifugation at 4°C and 8000 ***g*** for 10 min. This was done using a high‐speed centrifuge with a cooling system (GYTD‐4, Xiamen Guoy, China). Subsequently, the upper liquid was gathered and transferred to a separatory funnel for a two‐phase separation. The precise measurements and recording of volumes were conducted once the complete separation of the two phases occurred, with the top phase being notably abundant in anthocyanin (Yue et al., 2024). Liu et al. (2013) defined the extraction efficiency (E) as the ratio of the anthocyanin content in the upper phase to the overall anthocyanin content found in the onion peel, as demonstrated in the formula provided below (Liu et al., 2013).

$$E=C_{t}\times \frac{V_{t}}{M_{t}}$$

*C*_*t*_ represents the anthocyanin concentration in the upper layer, while *V*_*t*_ denotes the volume of this upper layer. *M*_*t*_ is the total anthocyanin content, which includes the sum from both the top and bottom layers.

### 2.11 Determination of total anthocyanin contents

Quantification was performed using the differential pH method (AOAC, 2005), employing a spectrophotometer for measurement (PG‐instrument Ltd, USA). The pH differential method was used to measure the anthocyanin content. Anthocyanin shows different colors depending on the pH: it appears reddish at pH 1 and becomes colorless at pH 4.5. Therefore, the samples were diluted in buffers at pH 1 and pH 4.5. For each test, a 0.1 mL sample was mixed with 1.4 mL of each buffer solution and then vortexed. The absorbance of these mixtures was measured at wavelengths of 510 and 700 nm using a spectrophotometer. These data were used to calculate the anthocyanin content with the specified formula:

$$\text{Anthocyanin}\;\left(\mathrm{mg}/L\right)=\frac{A\times 1000\times \mathrm{MW}\times \mathrm{DF}}{\epsilon \times L}$$

where *L* represents the path length of the cell, set at 1 cm, and *A* is calculated as the difference between absorbance at 510 and 700 nm at pH 1, subtracted by the difference in absorbance at the same wavelengths at pH 4.5. MW refers to the molecular weight of the standard anthocyanin, DF denotes the dilution factor, l indicates the path length in centimeters, ε is the molar extinction coefficient expressed in liters per mole per centimeter, and the number 1000 is used to convert the unit from grams to milligrams (Kang et al., 2021).

### 2.12 Identification of anthocyanin by LC–MS

Liquid chromatography‐mass spectroscopy (Agilent 1200‐6410, USA) was used to isolate and identify anthocyanin compounds. This system was equipped with a C18 column, a pump connected to a photo diode array (PDA), and a linear trap quadripole (LTQ) mass spectrometer. In order to separate and identify anthocyanin compounds, two mobile phases, including phase A (0.1% volume – volume solution of formic acid in water) and phase B (0.1% volume – volume of formic acid in acetonitrile), were used. The flow rate was 200 μL/min and the mentioned phases were used in gradient mode as follows: 2% B zero minutes, a linear growth in the concentration of B from 2% to 20% is observed over the period from 0 to 70 min, followed by a linear rise in B concentration from 20% to 80% during the time span from 70 to 100 min, a linear increase of B concentration from 80% to 98% during 100.1–100 min, a constant concentration of 98% for B during 110–100.1 min, a reduction of B amount to 2% during 110.1–110 min, and finally, a constant concentration of 2% during 110.1–120 min. After the isolation of bioactive compounds, mass scanning in the range of 100–2000 m/z and in positive ion mode was used to identify the compounds.

The identification and quantification of compounds were carried out by matching their mass spectrometry data with that of reference standards, database entries, or information sourced from past literature.

### 2.13 Statistical analysis

Every experiment was conducted a minimum of two times, and measurements were taken at least three times. Data statistical analysis was done using Microsoft Excel. To identify any significant differences between treatments, an analysis of variance was conducted using the SPSS program, setting a confidence level of .05.

## 3 RESULTS AND DISCUSSION

### 3.1 Extraction efficiency

**FIGURE 1:**
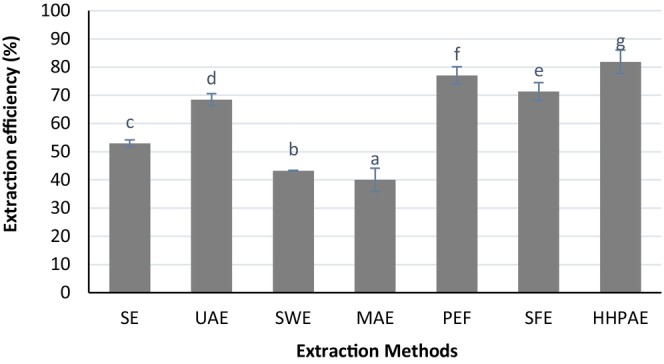
Extraction efficiency (%) with different extraction methods: solvent extraction (SE), ultrasound‐assisted extraction (UAE), subcritical water extraction (SWE), microwave‐assisted extraction (MAE), pulsed electric field (PEF) extraction, supercritical fluid extraction (SFE), and High hydrostatic pressure‐assisted (HHPAE) extraction. Significant differences (*p* < .05) are highlighted with different letters.

Figure 1 illustrates the comparative effectiveness of various extraction techniques. According to the shape, HHPAE showed the highest efficiency, and MAE showed the lowest efficiency. Regarding other extraction methods, PEF, SFE, UAE, SE, and SWE had the highest extraction efficiency, respectively.

The extraction yield of bioactive components is influenced by a variety of factors, including the nature of the bioactive compound, raw material characteristics, particle size, solvent selection, solvent‐to‐material ratio, extraction method, temperature, extraction time, pressure (if applicable), pH level, and safety and environmental considerations. Optimizing these factors based on the specific characteristics of the bioactive compound and the raw material can help achieve a higher extraction yield and produce extracts with better overall quality.

High‐pressure extraction can be performed at relatively lower temperatures compared to traditional methods, reducing the risk of thermal degradation of heat‐sensitive bioactive compounds such as anthocyanin. Hence, the ability to operate at lower temperatures reduces the risk of thermal degradation, preserving the integrity and bioactivity of thermally sensitive compounds during the extraction process. In addition, high‐pressure extraction methods often require shorter extraction times, leading to increased efficiency and reduced energy consumption. This is particularly advantageous for the extraction of labile compounds. The increased solubility of compounds at high pressures improves mass transfer and extraction efficiency. This results in higher yields of bioactive components from the raw material. Supercritical fluid extraction, in particular, is considered environmentally friendly since it often eliminates the need for organic solvents. Supercritical carbon dioxide is non‐toxic, non‐flammable, and easily recoverable, making it a safer and greener option. High‐pressure extraction methods, especially those using supercritical fluids, can produce extracts with lower residual solvent levels compared to traditional solvent‐based extraction methods.

Microwave‐assisted extraction (MAE) is a modern extraction technique that utilizes microwave energy to enhance the extraction of bioactive components from various raw materials. While MAE has several advantages, it also comes with certain disadvantages compared to other extraction methods. Microwave energy tends to selectively heat certain components based on their dielectric properties. This can lead to uneven heating within the sample, potentially causing the degradation of heat‐sensitive bioactives such as anthocyanin compounds or uneven extraction. Microwaves have limited penetration depth in certain materials, which may result in the incomplete extraction of bioactive compounds from deep within the sample. This limitation can affect the overall efficiency of the extraction process.

Several studies have been done on the comparison of different extraction methods, their advantages and disadvantages, and their optimization. Hannachi et al. (2019) found that the use of microwave‐assisted extraction (MAE) and ultrasound‐assisted extraction (UAE) surpassed the efficiency of traditional heat‐reflux extraction for the extraction of polyphenols from olive leaves. One significant benefit of these modern techniques is their lower heating of the biomaterial in comparison to conventional extraction (CE). This aspect is crucial because numerous organic compounds, such as anthocyanins, are heat‐sensitive. Prolonged exposure to high temperatures can cause these compounds to denature, reduce their biological effectiveness, or alter into other substances (Hannachi et al., 2019). Similarly, Drinić et al. (2021) assessed and evaluated different extraction techniques, such as soxhlet extraction, hydrodistillation, subcritical water extraction, and a dual process involving supercritical carbon dioxide extraction followed by traditional extraction, for obtaining bioactive compounds from the aerial parts of industrial hemp (*Cannabis sativa* L.). The results showed that the combined approach of supercritical carbon dioxide extraction preceding conventional extraction emerged as the most efficient technique for extracting bioactive compounds from hemp. (Drinić et al., 2021). Rajha et al. (2019) conducted a study comparing the effectiveness of different extraction methods for obtaining bioactive compounds from pomegranate peels. The methods compared included conventional extraction (CE), infrared irradiation‐assisted extraction (IR), ultrasound‐assisted extraction (US), pulsed electric fields (PEF), and high‐voltage electrical discharges (HVED). Their findings revealed that the HVED‐assisted extraction method significantly improved the recovery of polyphenols, approximately three times more than US and 1.3 times more than PEF. The analysis using scanning electron microscopy (SEM) suggested that the enhanced yield of total polyphenols after HVED (High Voltage Electric Discharge) treatment can be attributed to significant damage observed in the microstructure of the pomegranate skins (Rajha et al., 2019).

### 3.2 Total anthocyanin content

**FIGURE 2:**
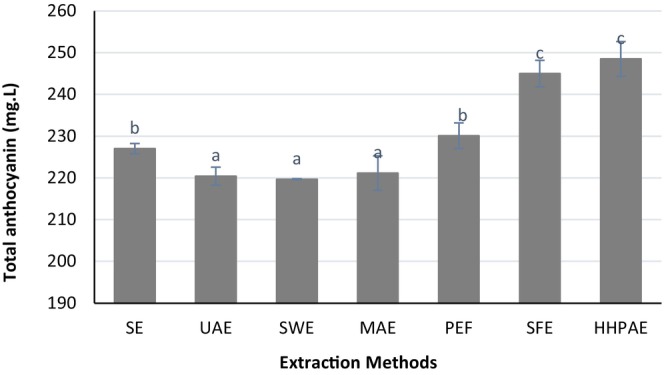
Total anthocyanin content extracted by different extraction methods: solvent extraction (SE), ultrasound‐assisted extraction (UAE), subcritical water extraction (SWE), microwave‐assisted extraction (MAE), pulsed electric field (PEF) extraction, supercritical fluid extraction (SFE), high hydrostatic pressure‐assisted (HHPAE) extraction. Significant differences (*p* < .05) are highlighted with different letters.

The results of total monomeric anthocyanin content using different extraction methods are depicted in Figure 2. SFE and HHPAE yielded the highest amounts of anthocyanin, whereas UAE, SWE, and MAE resulted in the lowest amounts. Additionally, no significant difference was observed between PEF and SE methods (*p* > .05).

Innovative extraction methods offer advantages such as reduced time requirements, minimal solvent usage, eco‐friendliness, sustainability, operation within a low‐temperature range, and high extraction yields. However, these methods also come with drawbacks, including high equipment costs, limited scalability, and challenges in industrial applications (Wani et al., 2021). The appropriateness of a given extraction technique for a certain pigment is influenced by multiple variables. For example, pigments that are polar (soluble in water) might be adversely affected by extraction methods that utilize electric fields, such as PEF and MEF. Likewise, temperature management is vital in methods like ultrasound (US) and MEF, as they can lead to unintended rises in temperature. Consequently, pressure‐driven technologies are better matched for pigments sensitive to heat, whereas methods based on electric fields are preferable for extracting non‐polar pigments (Islam et al., 2023). Emerging extraction techniques, such as HHPAE and SFE, tackle these challenges effectively. Specifically, SFE concentrates on breaking down cells, increasing membrane permeability, and boosting the transfer of intracellular fluids and components from vacuoles. The benefits of this approach include a higher yield of extraction, shorter processing times, streamlined purification of extracts, less dependence on traditional extraction variables, minimized degradation of compounds sensitive to heat, and lower energy expenses and environmental footprint (Usman et al., 2022).

PEF technology, by promoting electroporation in cell membranes, improves the diffusion of important plant substances at room temperature. This low‐temperature extraction process facilitated by PEF helps in preserving the cell wall integrity and ensures the efficient extraction of components such as anthocyanin and other compounds into the solvent. Contrary to extraction methods that involve high temperatures and potentially speed up chemical processes (such as the Maillard reaction, which can alter color), PEF extraction is apt for obtaining pure substances including dry matter, carotenoids, vitamins, sucrose, proteins, and inulin (Chhikara et al., 2023; Wang et al., 2018).

Gachovska et al. (2010) evaluated the effect of pulsed electric field (PEF) processing on anthocyanin extraction from red cabbage using water as the extraction medium. Their findings demonstrated that PEF processing enhanced the overall yield of anthocyanins extracted in water by a factor of 2.15, with a greater amount of nonacylated anthocyanins relative to the untreated samples, indicating statistical significance (*p* < .05) (Gachovska et al., 2010).

Velarde‐Salcedo et al. (2023) applied supercritical fluid extraction (SFE) and maceration to obtain the essential oil from raspberry waste. They reported that SFE allowed the extraction of compounds not reported before in red raspberry, such as pinocembrin and farnesol.

Pereira et al. (2016) compared the antioxidant capacity and bioactive components of Portuguese myrtle (*Myrtus communis* L.) by supercritical fluid extraction vs. conventional extraction. Extracts obtained through supercritical fluid extraction (SFE) were produced at a pressure of 23 MPa and a temperature of 45°C, with a carbon dioxide flow rate of 0.3 kg/h and ethanol used as a co‐solvent at a flow rate of 0.09 kg/h. Hydrodistillation was performed using a Clevenger‐type apparatus, and the resulting aqueous phase was extracted using diisopropylether, resulting in the production of what is referred to as the Liquid Phase Extract (LPE). The findings from this study indicate that the yields from supercritical fluid extraction (SFE) of myrtle are superior to those obtained through liquid‐phase extraction (LPE) (Pereira et al., 2016).

Ultrasound‐assisted and subcritical water extraction techniques were applied for maximal recovery of phenolic compounds from raw ginger herbal dust by Sulejmanović et al. (2024). SWE showed a higher yield than UAE, and more polyphenols were extracted by UAE (Sulejmanović et al., 2024).

Vladić et al. (2020) utilized both SWE and MAE to extract phenolic compounds from pomegranate peel. When comparing these two methods, it was found that MAE was more effective than SWE in extracting phenolics from pomegranate peel, and it also resulted in extracts free of hydroxymethylfurfural (HMF) (Vladić et al., 2020).

In general, no study has been conducted on the effect of new and green extraction methods on the amount of anthocyanin extracted from plant compounds. Also, the difference between different methods in different articles depends on the nature of the substance and the extraction method. However, the obtained results are in accordance with the results of previous studies and show that methods such as HHPAE and SFE led to the preservation of more anthocyanin and higher efficiency due to less degradation of sensitive compounds such as anthocyanin.

### 3.3 Anthocyanin identification

**FIGURE 3:**
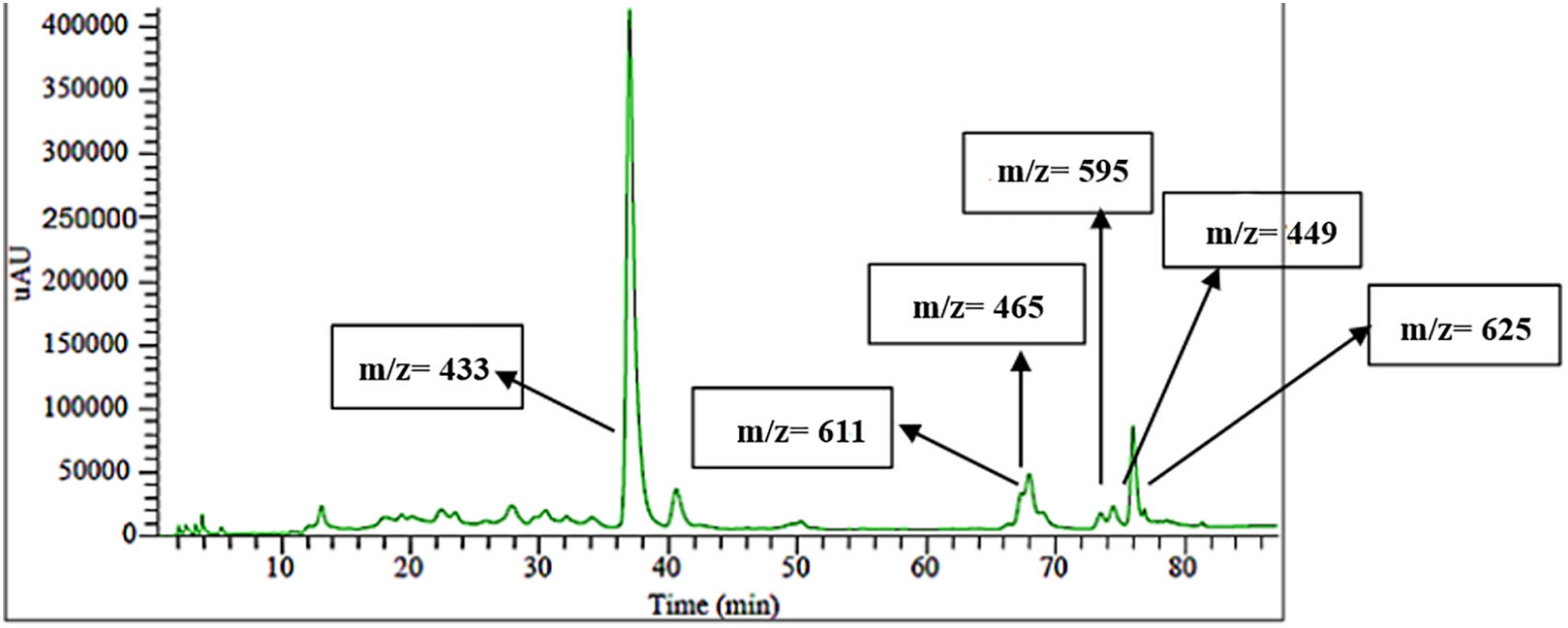
The chromatogram of the anthocyanin extraction from red onion skin.

**FIGURE 4:**
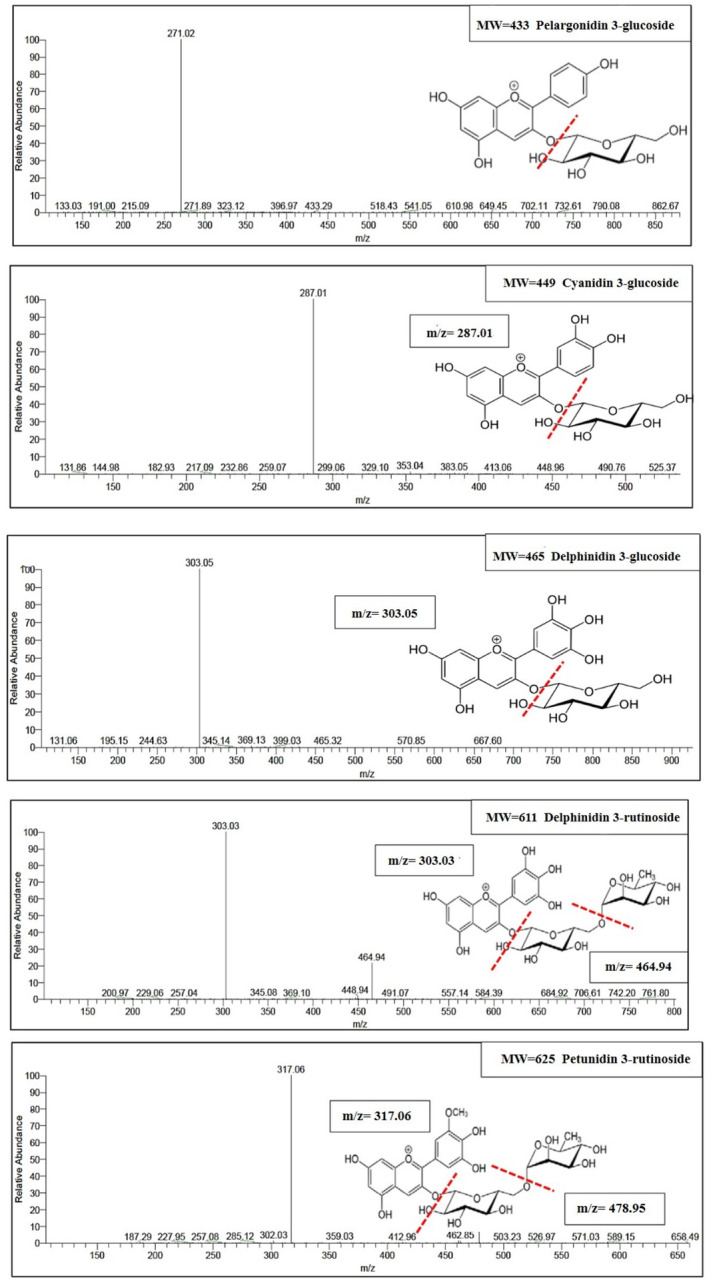
MS/MS mass spectrum of anthocyanin compounds of red onion skin.

Anthocyanins are composed of anthocyanidins, also known as aglycones, which are bonded to sugars to form glycosides. Anthocyanidins typically have a sugar molecule attached at the 3‐position, and this sugar component can be acylated with either aliphatic or aromatic acids. In nature, approximately 17 anthocyanidins have been identified. However, over 90% of the anthocyanins isolated from natural sources are derived from just six anthocyanidins: pelargonidin, cyanidin, peonidin, delphinidin, petunidin, and malvidin (Babaloo & Jamei, 2018). The red onion skin contains four main types of anthocyanins, including delphinidin, cyanidin, and pelargonidin and petundin which bond with sugar to form glycosidic compounds (Figures 3 and 4). The mass spectrum shows a compound with a molecular mass of 433, whose MS/MS spectrum has an ion component of m/z 271. The ionic component m/z 271 belongs to Pelargonidin, and the reduction of 162 mass units is related to a hexose molecule, so according to the results of other studies, it can be concluded that this compound is probably Pelargonidin 3‐glycoside. Similarly, other mass spectra were also checked, and based on Figure 4, it was determined that the most common anthocyanins found in red onions include petunidin 3‐rutinoside, delphinidin 3‐rutinoside, cyanidin‐3‐rutinoside, delphinidin 3‐glucoside, cyanidin 3‐glucoside, and pelargonidin 3‐glucoside. The main anthocyanin in the extraction from red onion was consistent with previous studies (Fossen & Andersen, 2003; Oancea & Drăghici, 2013; Samota et al., 2022; Zhang et al., 2016).

## 4 CONCLUSIONS

This study highlights the potential of green extraction methods for efficiently extracting and purifying anthocyanins from red onion skin. As traditional extraction methods pose environmental challenges, developing eco‐friendly alternatives is crucial. Our research explored various novel extraction techniques alongside solvent extraction for anthocyanin extraction from red onion peel, a significant food industry waste. Our findings demonstrate that high‐pressure processing and pulsed electric field methods were particularly effective, yielding high anthocyanin quantities with notable efficiency. Despite their capability to enhance extraction yield and extract stability, these technologies necessitate advanced instrumentation, increasing production costs. Nevertheless, contemporary eco‐friendly methods such as high‐pressure processing and pulsed electric fields offer promising solutions for anthocyanin extraction from food by‐products like red onion skins, maintaining production quality while potentially reducing costs. Future research should focus on developing cost‐effective machinery and implementing safer, customized procedures. Comprehensive investigations into both extraction methods and their specific applications are essential to ensure sustainable product development. These efforts will promote the wider adoption of green technologies in the food industry, fostering environmental sustainability and resource efficiency.

## AUTHOR CONTRIBUTIONS

**Nasim Mirzazadeh:** Formal analysis (equal); investigation (equal); writing – original draft (equal). **Hadiseh Bagheri:** Data curation (equal); funding acquisition (equal); methodology (equal); writing – original draft (equal). **Somaye Soleimanimehr:** Formal analysis (equal); investigation (equal); writing – review and editing (equal). **Fatemeh Rasi:** Data curation (equal); methodology (equal); writing – review and editing (equal). **Sahar Akhavan‐Mahdavi:** Conceptualization (lead); project administration (lead); supervision (lead); validation (lead); visualization (equal).

## FUNDING INFORMATION

None.

## CONFLICT OF INTEREST STATEMENT

The authors declare no conflict of interest relevant to this article.

## Data Availability

The data that support the findings of this study are available on request from the corresponding author. The data are not publicly available due to privacy or ethical restrictions.
